# Disordered Eating and Lifestyle Studies—2nd Edition

**DOI:** 10.3390/nu16203450

**Published:** 2024-10-11

**Authors:** Sónia Gonçalves, Bárbara Cesar Machado

**Affiliations:** 1 Psychology Research Center (CIPsi), School of Psychology, University of Minho, 4710-057 Braga, Portugal; sgoncalves@psi.uminho.pt; 2 Research Centre for Human Development (CEDH), Faculty of Education and Psychology, Universidade Católica Portuguesa, 4169-005 Porto, Portugal

Disordered eating, eating disorders, and lifestyle are complex and interconnected topics that intersect with various aspects of physical health, mental well-being, and societal influences. Disordered eating refers to a broad range of abnormal eating behaviors that may not meet the clinical criteria for a diagnosed eating disorder but can still negatively impact both physical and psychological health. Examples of disordered eating behaviors include restrictive eating, binge eating, compulsive overeating, and an excessive focus on food and body image. These behaviors can be influenced by numerous factors, including genetics, psychological factors, social pressures, and cultural norms. Addressing disordered eating early is crucial to preventing the development of full-blown eating disorders and promoting a healthy relationship with food.

Eating disorders, on the other hand, are serious mental health conditions characterized by significant disturbances in eating behaviors, perceptions of body weight and shape, and emotional regulation. The most common eating disorders include anorexia nervosa, bulimia nervosa, and binge eating disorder. These conditions can have severe physical health consequences, including malnutrition, electrolyte imbalances, and damage to vital organs. Furthermore, eating disorders often co-occur with other mental health conditions such as depression, anxiety, and substance use disorders.

Recent research has shed light on the complexities and underlying mechanisms of disordered eating and eating disorders. For example, studies have revealed significant neurobiological components to these conditions, including alterations in brain structure and function related to reward processing, impulse control, and emotional regulation [1]. Genetic research has also progressed, identifying specific genetic variants associated with an increased susceptibility to eating disorders [2]. These findings underscore the importance of considering both biological predispositions and environmental factors when understanding and addressing these conditions.

Lifestyle factors also play a significant role in shaping eating behaviors, body image, and overall health outcomes. Societal pressures, media portrayals of body ideals, and cultural attitudes towards food can all influence individuals’ lifestyle choices and attitudes toward eating. Healthy lifestyle practices, including balanced nutrition, regular physical activity, stress management, and adequate sleep, are essential for maintaining overall well-being and preventing disordered eating and eating disorders. Therefore, promoting positive lifestyle changes that prioritize self-care, body positivity, and mental health support is of utmost importance.

In this special issue, we present several studies that focus on specific conditions such as orthorexia nervosa and inflammatory bowel disease [3], as well as orthorexia in specific populations like physically active adults [4] and military flying personnel [5]. These studies aim to enhance our understanding of orthorexia nervosa and highlight the need for further research into this condition, which appears to be less studied compared to other forms of disordered eating, such as restriction and purging. Orthorexia nervosa seems to be particularly common among certain populations, such as men and physically active adults. We believe that this form of disordered eating could be closely related to specific lifestyles, and more research is needed to clarify the continuum between healthy eating habits and disordered eating with significant clinical impairment.

This special issue also includes a study on the impact of the COVID-19 pandemic from the perspective of adolescents with anorexia nervosa [6]. The findings emphasize that the pandemic-related confinement had a negative effect on anorexia nervosa symptoms. This underscores the need for more studies on the impact of COVID-19 on our lives, particularly how the lockdown has altered our behaviors and overall well-being.

Another study in this issue examines social eating behavior, family meals, and disordered eating in adolescence [7]. The study concludes that family meals and social eating behaviors are inversely associated with disordered eating in adolescence, highlighting the potential protective effect of regular social and family meals in preventing disordered eating.

Finally, this special issue includes a significant contribution in the form of a systematic review that explores the relationship between loss of control, reward sensitivity, and eating behaviors in children and adolescents [8]. Despite the scarcity of studies in this area, the review emphasizes the need for further research to raise awareness of these emerging concepts.

There remain significant gaps in our knowledge, particularly regarding the prevention and early intervention of disordered eating behaviors. While it is well established that early intervention can significantly improve outcomes, there is still a lack of consensus on the most effective strategies for identifying and addressing disordered eating behaviors before they develop into full-blown eating disorders [9]. Additionally, more research is needed to understand the long-term efficacy of various treatment approaches, including psychotherapy, pharmacotherapy, and lifestyle interventions [10].

Future research should focus on several key areas to address these gaps. Firstly, there is a need for longitudinal studies that track individuals from early childhood through adulthood to identify early markers of disordered eating and eating disorders and to understand how these behaviors develop over time. Such studies could provide valuable insights into the most effective points for intervention. Secondly, research should explore the impact of emerging digital and social media platforms on body image and eating behaviors, given the increasing influence of these platforms on individuals’ lives [11]. Understanding how to mitigate the negative impacts of these platforms while leveraging their potential for positive messaging could be crucial for prevention efforts.

Moreover, there is a need to develop and evaluate comprehensive prevention programs that integrate education about healthy eating, body positivity, and media literacy into school curricula. These programs should be culturally sensitive and tailored to the needs of diverse populations to ensure they are effective across different demographic groups [12]. Finally, future research should also examine the role of community and family-based interventions in preventing and treating disordered eating and eating disorders. Engaging families and communities in these efforts can provide a supportive environment that reinforces positive behaviors and attitudes towards food and body image.

In summary, disordered eating and lifestyle are interconnected issues that require a comprehensive approach involving education, prevention, early intervention, and support for individuals affected by these conditions. By raising awareness, promoting positive body image, and addressing societal influences, we can work towards fostering a healthier relationship with food, body image, and overall well-being. Future research should continue to explore the underlying mechanisms of disordered eating and eating disorders, develop effective prevention and intervention strategies, and leverage the power of community and family support to create lasting change.

In the special issue “Disordered Eating and Lifestyle Studies II,” we aimed to address the complexities surrounding disordered eating behaviors, eating disorders, and their relationship and impact on lifestyle dynamics. The bidirectional relationship between disordered eating and lifestyle factors is also explored and highlighted.
